# Assessment of Cardiorenal Involvement in Systemic Sclerosis Patients

**DOI:** 10.3390/biom15091297

**Published:** 2025-09-09

**Authors:** Chiara Pellicano, Giancarlo D’Ippolito, Annalisa Villa, Ottavio Martellucci, Umberto Basile, Valeria Carnazzo, Valerio Basile, Edoardo Rosato, Mariapaola Marino, Antonietta Gigante

**Affiliations:** 1 Department of Translational and Precision Medicine, La Sapienza University of Rome, Piazzale Aldo Moro 5, 00185 Rome, Italy; chiara.pellicano@uniroma1.it (C.P.); giancarlo.dippolito@uniroma1.it (G.D.); annalisa.villa@uniroma1.it (A.V.); ottavio.martellucci@uniroma1.it (O.M.); edoardo.rosato@uniroma1.it (E.R.); 2 Dipartimento di Patologia Clinica, Ospedale Santa Maria Goretti, A.U.S.L. Latina, 04100 Latina, Italy; u.basile@ausl.latina.it (U.B.); v.carnazzo@ausl.latina.it (V.C.); 3 Clinical Pathology Unit and Cancer Biobank, Department of Research and Advanced Technologies, I.R.C.C.S. Regina Elena National Cancer Institute, 00144 Rome, Italy; valeriobasile90@gmail.com; 4 Sezione di Patologia Generale, Dipartimento di Medicina e Chirurgia Traslazionale, Università Cattolica del Sacro Cuore, 00168 Rome, Italy; mariapaola.marino@unicatt.it; 5 Sezione di Patologia Generale, Dipartimento di Medicina e Chirurgia Traslazionale, Fondazione Policlinico Universitario “A. Gemelli” I.R.C.C.S., 00168 Rome, Italy

**Keywords:** systemic sclerosis, pulmonary arterial hypertension, chronic kidney disease, cardiorenal syndrome

## Abstract

Systemic sclerosis (SSc) is an autoimmune disease associated with a high burden of morbidity and mortality due to organ complications. Pulmonary arterial hypertension (PAH) and cardiac involvement, characterized by chronic right ventricular (RV) pressure overload with consequent RV dysfunction and ultimately right heart failure (HF), are among these. A common comorbidity in SSc is chronic kidney disease (CKD). CKD is often present at the time of PAH diagnosis or a decline in renal function may occur during the course of the disease. CKD is strongly and independently associated with mortality in patients with PAH and HF. The cardiovascular and renal systems are closely interconnected, and disruption of this balance may result in cardiorenal syndrome (CRS). Type 2 CRS refers to CKD as a consequence of chronic HF. In clinical practice, non-specific markers such as troponin, B-type natriuretic peptide (BNP), N-terminal pro-BNP (NT-proBNP), and serum creatinine aid in CRS diagnosis. More specific biomarkers, including cystatin C (CysC), neutrophil gelatinase-associated lipocalin (NGAL), galectin-3, and soluble urokinase plasminogen activator receptor (suPAR), have shown value for diagnosis and prognosis in CRS. This study aimed to evaluate comprehensively heart/kidney damage markers related to CRS in SSc patients compared with healthy controls (HC) and to examine their association with renal and cardiac ultrasound parameters. SSc patients showed significantly higher CRS markers than HC (*p* < 0.001). SSc patients with clinically diagnosed CRS had significantly elevated galectin-3, suPAR, sNGAL, and uNGAL levels (*p* < 0.05) than SSc patients without CRS. Positive correlations were found between renal resistive index (RRI) and NT-proBNP (r = 0.335, *p* < 0.05), and between RRI and suPAR (r = 0.331, *p* < 0.05). NT-proBNP, suPAR, galectin-3, sNGAL, and uNGAL emerge as promising biomarkers for the early detection of cardiac and renal involvement in SSc patients.

## 1. Introduction

Systemic sclerosis (SSc) is a systemic autoimmune disease characterized by microvascular changes, dysregulation of the immune system, and fibrosis. It affects multiple organs such as skin, lungs, heart, kidneys, musculoskeletal system, and gastrointestinal tract [1]. The cardiovascular and renal systems are interdependent, and their delicate balance, when disrupted, can lead to the development of cardiorenal syndrome (CRS) [2]. CRS is determined by symptoms affecting both heart and kidneys; the reduced cardiac output is responsible of a renal hypoperfusion leading to the activation of renin–angiotensin–aldosterone system (RAAS), the sympathetic nervous system (SNS), and the release of vasopressin (ADH), inducing fluid retention and volume overload [3]. Furthermore, the heart failure (HF) causes reduced venous return and subsequent renal venous congestion determining a reduction in glomerular filtration rate (GFR) [2,4].

In clinical practice, the diagnosis of CRS is supported by cardiac and renal biomarkers such as troponin, B-type natriuretic peptide (BNP) or N-terminal pro-B-type natriuretic peptide (NT-proBNP), serum creatinine (sCr) and GFR. Specific biomarkers for the diagnosis of CRS have not been established yet, therefore the evaluation of biomarkers for acute kidney injury (AKI), including cystatin C (CysC), neutrophil gelatinase-associated lipocalin (NGAL), galectin-3 and soluble urokinase plasminogen activator (suPAR), is becoming more and more important at both diagnosis and prognosis levels.

CysC is a protein normally produced by all the nucleated cells and is fully filtered and completely reabsorbed by the kidneys, representing an excellent biomarker of GFR. Moreover, CysC increases earlier than creatinine which is affected by physiological processes and rises only when at least 50% of the kidney is involved [5,6]. We previously demonstrated a greater statistically significant positive correlation between renal resistive index (RRI) and a greater statistically significant negative correlation between RRI and eGFR_CysC_ in a cohort of SSc patients [7].

NGAL is a protein produced by cardiomyocytes and renal cells which levels are influenced by ischemic and toxic AKI. In addition, it is associated with adverse outcomes in patients with chronic HF and good renal function [8].

Galectin-3, instead, is a protein produced by macrophages during inflammation. It is involved in tissue remodeling and, furthermore, increases in patients with AKI and appears to be predictable of CRS [9].

Finally, suPAR is the soluble form of the urokinase plasminogen activator receptor (uPAR) produced and released into the bloodstream by inflammatory cells. Its levels increase during acute and chronic systemic inflammation, and its serum concentration is associated with worsening renal function and a poorer prognosis regardless of GFR value. It appears that kidneys and heart are involved in the clearance of suPAR, hypothesizing a central role of this receptor in the pathogenesis of cardiac disease [10].

However, despite the growing interest in the field, validated markers for CRS are still lacking.

In the context of SSc, where cardiac and renal involvement are both common and impact outcomes, a comprehensive evaluation of CRS-specific biomarkers is a current unmet need.

The aim of this study is to evaluate collectively the markers of heart/kidney damage most closely related to CRS, such as galectin-3, serum and urinary NGAL (sNGAL and uNGAL) and suPAR, in patients with SSc and to evaluate a possible association with renal and cardiac ultrasound parameters.

## 2. Materials and Methods

### 2.1. Study Population

A total of 76 consecutive SSc patients were included in the study, all referred to the Regional Reference Center of Policlinico Umberto I with a diagnosis of SSc according to the 2013 ACR/EULAR criteria [11]. Fifty healthy donors, matched by sex and age with the study group were also enrolled. Inclusion criteria were as follows: age ≥ 18 years and ability to provide written informed consent. Exclusion criteria were as follows: cardiovascular and pulmonary diseases not related to SSc, pulmonary arterial hypertension (PAH), liver failure, renal failure, scleroderma renal crisis (SRC), glomerulonephritis, urinary infections, renal artery stenosis, diabetes, systemic arterial hypertension, thyroid dysfunction, neoplastic diseases. Smoking, pregnancy, breastfeeding, and treatment within the past 6 months with immunosuppressive agents or corticosteroids at a dose ≥ 10 mg/day of prednisolone were also part of the exclusion criteria. Moreover, patients were treated with angiotensin-converting enzyme inhibitors and angiotensin II receptor blockers. All enrolled patients received standard treatments, including calcium channel blockers and iloprost infusions, as part of their care.

The study was conducted in accordance with the Declaration of Helsinki, and all participants provided written informed consent. The study was approved by the Ethics Committee of Sapienza University of Rome (EC n 0304).

### 2.2. Clinical Assessment

All patients underwent evaluation of the main clinimetric disease indices.

The extent of skin involvement and differentiation between diffuse (dcSSc) or limited (lcSSc) form of disease were assessed using the modified Rodnan skin score (mRSS) [12].

Disease activity level was measured using the Disease Activity Index (DAI) [13], which includes six variables assigned a numerical score: change in skin symptoms in the past month, presence of digital ulcers, mRSS ≥ 18; presence of tendon friction rubs, C-reactive protein (CRP) ≥ 1 mg/dL; diffusing lungs for carbon monoxide (DLco) ≤ 70% of predicted value.

Disease severity was assessed using the Disease Severity Scale (DSS) [14], which is based on nine domains: general condition, peripheral vessels, skin, joints/tendons, muscles, gastrointestinal tract, lungs, heart, and kidneys. Each organ or system is scored separately from 0 to 4, considering absent, mild, moderate, severe, or “end-stage” involvement.

The demographic and clinical profiles of the enrolled patients have been reported in a previous study [7].

### 2.3. Nailfold Videocapillaroscopy

Nailfold videocapillaroscopy (NVC) was performed in all patients at the distal phalanges of the second, third, and fourth fingers of both hands. The examination was conducted using a 200× magnification capillaroscope (VideoCap 3.0, DS Medica, Milan, Italy), after 20 min of acclimatization in a room at a controlled temperature of 24 ± 0.4 °C.

According to Cutolo et al. [15], the capillaroscopic patterns were classified as early, active, and late.

### 2.4. Renal Ultrasound and Intrarenal Arteries Doppler Ultrasonography

All patients were examined after at least 15 min of acclimatization in a room with controlled temperature at 22 ± 0.4 °C. Patients on intravenous prostacyclin therapy were examined the day before their next infusion. Renal diameters and Doppler indices were measured by the same experienced nephrologist. The operator performing the Doppler study was blinded to the clinical characteristics of the patients. Measurements were taken with the patient in the supine position, who was asked to hold their breath during the exam. Renal color Doppler ultrasound was performed using a Toshiba Aplio Ultrasound System SSA-790 (Tokyo, Japan) with a convex 3.5 MHz probe. The Doppler signal was always assessed with an angle of less than 60°. Doppler indices were measured in both kidneys at the level of the intrarenal arteries (upper pole, mid-kidney, and lower pole). The mean of the three measurements was selected as the final value for each kidney. An anterior approach was used to identify the origin of the renal arteries, and an oblique lateral approach was used for the intermediate and intrarenal segments.

The following renal ultrasound parameters were measured: renal diameter, parenchymal thickness, renal sinus, and atrophy index. Longitudinal renal diameter was measured bilaterally as the greatest distance between the upper and lower poles in the sagittal plane. Parenchymal thickness was obtained at least at three different points as the shortest distance from the renal sinus to the renal capsule. The atrophy index was calculated as the ratio of longitudinal renal diameter to the maximum diameter of the renal sinus.

The following Doppler indices were measured: peak systolic velocity (PSV), end diastolic velocity (EDV), renal resistive index (RRI), pulsatile index (PI), and systolic/diastolic ratio (S/D). RRI was calculated as (peak systolic frequency shift − minimum diastolic frequency shift)/peak systolic frequency shift; PI was calculated as (peak systolic frequency shift − minimum diastolic frequency shift)/mean frequency shift. PSV and EDV were expressed in cm/s. The reference value for RRI in healthy adults was defined as 0.60 ± 0.10, with 0.70 as the upper limit of normal [16].

### 2.5. Laboratory Tests

Peripheral venous blood samples were collected in tubes and centrifuged at 1500× *g* (3000 rpm, Thermo Jouan CR3i multifunction, Thermofisher Scientific, Waltham, MA, USA) for 15 min at 20 °C. Serum samples were aliquoted into 1.5 mL Eppendorf tubes and stored at −80 °C until testing. Thawing of samples occurred once, with maintenance at room temperature, and immediate analysis thereafter. The analysis was conducted by an independent operator blinded to the clinical history of the samples, ensuring an unbiased assessment.

Serum CysC (sCysC), suPAR, sNGAL, uNGAL, troponin, NT-proBNP assays were performed on core technology behind ARCHITECT (ABBOTT Laboratories, Abbott Park Illinois, USA) of immunoassay and clinical chemistry analyzers utilizing chemiluminescent microparticle immunoassay (CMIA) technology for immunoassays and methods for chemical analysis. The manufacturer states the assays performance have satisfactory precision as the assays are designed to have a low coefficient of variation (CV).

Serum galectin-3 concentration was quantified utilizing the Simple Plex™ cartridge-based assay on the Ella™ platform (ProteinSimple, San Jose, CA, USA), following the detailed instructions provided by the manufacturer. ELLA is a platform based on microfluidic technology that allows the performance of automated enzyme-linked immunoassays without manual steps of operator. The only manual steps to handle are the dilution of samples and the loading of diluted samples and washing buffer into the cartridge. The calibration curve for each cartridge is generated by the manufacturer for each lot and the ELLA system acquires calibration-related parameters through the reading of the cartridge’s barcode. The fluorescent signals are read inside the ELLA instrument and used for quantification based on master calibration curves. The assay time is 72 min. The cartridges are available in a multi- or single-analyte format. We used 72-plex single-analyte cartridges for human galectin-3.

Thawed samples underwent a single assay process under blinded conditions, all within a solitary batch. Serum dilutions were executed when necessary, adhering strictly to the recommended procedures of the manufacturer.

### 2.6. Selection of Patients with Cardiorenal Syndrome

The classification of CRS was assessed in accordance with the guidelines of the Acute Dialysis Quality Initiative (ADQI) [17] and the American Heart Association [2]. In SSc patients, cardiac and renal involvement is linked to chronic vasculopathy, therefore we selected patients by applying validated non-invasive tools commonly used in clinical practice to assess heart and kidney damage. In details, early pulmonary vasculopathy was defined by echocardiographic signs of inversion of the systole/diastole ratio of the left ventricle (E/A ratio ≤ 1) and right heart dysfunction, expressed by a reduced ratio of echocardiography-derived tricuspid annular plane systolic excursion (TAPSE) and pulmonary arterial systolic pressure (sPAP) (TAPSE/sPAP ≤ 0.8 mm/mmHg), after ruling out other cardiac diseases [18]. Early renal involvement was defined by a reduction in eGFR, using serum CysC for calculation, or presence of proteinuria.

Among all SSc patients, those with CRS were selected based on the coexistence of heart and renal damage, considering the following parameters: TAPSE/sPAP ≤ 0.8 mm/mmHg or E/A ≤ 1, and eGFR ≤ 60 mL/min or proteinuria.

### 2.7. Statistical Analysis

Statistical analysis was performed using SPSS version 25.0 (Bioz, Los Altos, CA, USA). After assessment of normality, continuous variables were expressed as mean ± standard deviation (SD) or median and interquartile range (IQR), as appropriate. Categorical variables were expressed as absolute frequency and percentage (%). To assess differences between groups, Student’s *t*-test or the Mann–Whitney U test was used as appropriate. For multiple comparisons, Bonferroni corrections were applied. The chi-square test or Fisher’s exact test were used to assess differences between categorical variables, as appropriate. For bivariate correlations, two-tailed Pearson or Spearman correlation tests were used. Stepwise logistic regression analysis was used to evaluate the association between a dependent dichotomous variable (CRS) and continuous independent variables (NT-proBNP, suPAR, galectin-3, sNGAL, and uNGAL) which were significant at univariable analysis. Results were expressed as odds ratio (OR) and 95% confidence interval (95% CI).

A *p*-value ≤ 0.05 was considered significant.

## 3. Results

### 3.1. Demographic and Clinical Characteristics of SSc Patients

Of the 76 patients, 65 (85.5%) were women. The mean age was 57.8 ± 10.7 years and mean disease duration was 15.1 ± 10.12 years. Forty patients (52.6%) had dcSSc and thirty-six (47.4%) had lcSSc, with a mean mRSS of 16.9 ± 9.4. Thirty-five patients (46.1%) tested positive for Scl-70 (anti-topoisomerase I) antibodies, eighteen patients (23.7%) were positive for CENP-B (anti-centromere), five patients (6.6%) were positive for RNA polymerase III, one patient (1.3%) tested positive for Th/To, and seventeen patients (22.4%) were exclusively positive for ANA (antinuclear antibodies). Twelve patients (15.8%) showed an “Early” pattern on nailfold videocapillaroscopy, twenty-four patients (31.6%) had an “Active” pattern, and forty (52.6%) had a “Late” pattern.

Mean serum creatinine (sCr) was 0.83 ± 0.19 mg/dL and mean sCysC was 1.12 ± 0.34 mg/L (*p* < 0.001), with a mean eGFR_Cr_ of 79.82 ± 17.77 mL/min and mean eGFR_CysC_ of 72.02 ± 20.78 mL/min. Only 12 (15.8%) SSc patients had eGFR_Cr_ ≤ 60 mL/min, whereas 25 (35.9%) had eGFR_CysC_ ≤ 60 mL/min (*p* < 0.001).

The mean TAPSE/sPAP ratio was 0.80 ± 0.17 mm/mmHg, and the mean E/A ratio was 1.10 ± 0.44. Patients with TAPSE/sPAP ≤ 0.8 mm/mmHg were 36 (48%), while patients with E/A ≤ 1 were 38 (50.7%). The mean longitudinal kidney diameter was 104.66 ± 8.61 mm, and mean parenchymal thickness was 16.23 ± 2.49 mm. Mean RRI was 0.68 ± 0.05, and 24 (31.6%) SSc patients had RRI ≥ 0.70.

The demographic and clinical characteristics of the SSc patients are shown in Table 1 [7].

### 3.2. Comparative Analysis of Biomarkers Between SSc Patients and HC

Mean sCysC (1.12 ± 0.34 mg/L vs. 0.66 ± 0.23 mg/L, *p* < 0.001), suPAR (4.86 ± 1.69 ng/mL vs. 2.20 ± 0.43 ng/mL, *p* < 0.001), NT-proBNP (7.09 ± 13 pg/mL vs. 0.42 ± 0.16 pg/mL, *p* < 0.001), troponin (3.77 ± 3.41 ng/L vs. 1.92 ± 1.34 ng/L, *p* < 0.001), galectin-3 (6327.08 ± 2393.43 pg/mL vs. 2264.08 ± 535.51 pg/mL, *p* < 0.001) and urinary protein levels (7.03 ± 1.97 mg/dL vs. 5.51 ± 0.43 mg/dL, *p* < 0.001) were significantly higher in SSc patients compared to HC. Mean sNGAL levels were significantly lower in SSc patients compared to HC (41.12 ± 22.44 ng/mL vs. 48.54 ± 4.28 ng/mL, *p* < 0.001) whilst mean uNGAL levels were significantly higher in SSc patients compared to HC (106.86 ± 22.38 ng/mL vs. 55.85 ± 21.30 ng/mL, *p* < 0.001).

**Figure 1:**
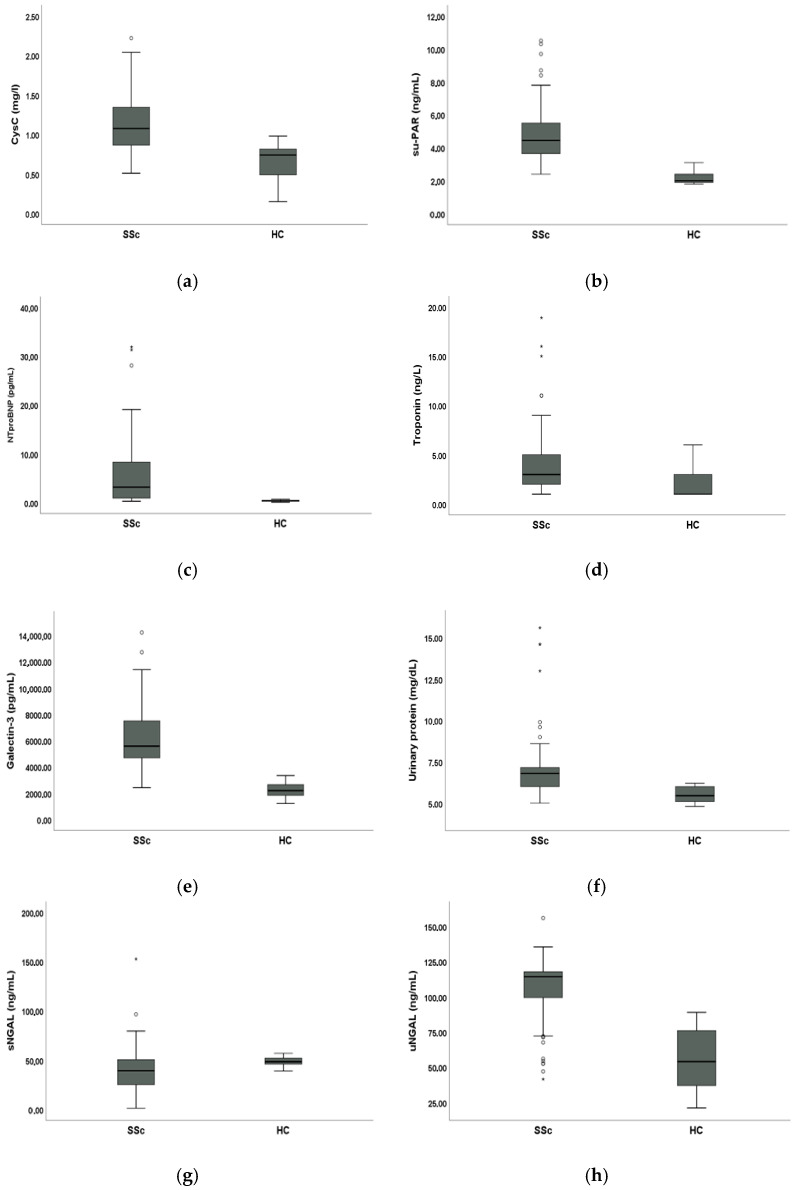
Comparative analysis of biomarkers between 76 systemic sclerosis (SSc) and 50 healthy controls (HC) enrolled: (**a**) Box plot showing median serum cystatin C (sCysC); (**b**) box plot showing median serum soluble urokinase plasminogen activator (suPAR); (**c**) box plot showing median serum N-terminal pro-B-type natriuretic peptide (NT-proBNP); (**d**) box plot showing median serum troponin; (**e**) box plot showing median serum galectin-3; (**f**) box plot showing median urinary protein; (**g**) box plot showing median serum neutrophil gelatinase-associated lipocalin (sNGAL); (**h**) box plot showing median urinary NGAL (uNGAL). Circles and stars are outliers.

These results are shown in Figure 1.

### 3.3. Analysis of Biomarkers in SSc Patients

There was a statistically significant positive linear correlation between RRI and NT-proBNP (r = 0.335, *p* < 0.05), and between RRI and suPAR (r = 0.331, *p* < 0.05).

There was a statistically significant positive linear correlation between NT-proBNP and suPAR (r = 0.245, *p* < 0.05), and between NT-proBNP and CysC (r = 0.350, *p* < 0.01).

There was a statistically significant positive linear correlation between galectin-3 and suPAR (r = 0.272, *p* < 0.05), and between galectin-3 and urinary proteins (r = 0.318, *p* < 0.01).

There was a statistically significant positive linear correlation between sNGAL and CysC (r = 0.293, *p* < 0.05).

There was also a statistically significant positive linear correlation between suPAR and uNGAL (r = 0.252, *p* < 0.05), and between suPAR and CysC (r = 0.315, *p* < 0.05).

We did not find any statistically significant correlation between NT-proBNP, troponin, suPAR, galectin-3, sNGAL, uNGAL and age, disease duration, mRSS, DAI and DSS among all SSc patients enrolled. Moreover, we did not find any statistically significant differences in the mean values of these parameters stratifying population according to sex and disease subset.

### 3.4. Comparative Analysis of Biomarkers Between SSc Patients with and Without CRS

Among SSc patients, those with CRS were selected based on the following parameters: TAPSE/sPAP ≤ 0.8 mm/mmHg or E/A ≤ 1, and eGFR_CysC_ ≤ 60 mL/min or proteinuria. One patient was excluded due to unavailable TAPSE data. The number of SSc patients with CRS was 16 (21.3%).

SSc patients with CRS had statistically significant higher mean NT-proBNP (12.36 ± 8.15 pg/mL vs. 5.79 ± 6.83 pg/mL, *p* < 0.05), mean galectin-3 (7349.69 ± 2088.51 pg/mL vs. 6049.76 ± 2411.23 pg/mL, *p* < 0.05), mean suPAR (5.35 ± 1.26 ng/mL vs. 4.70 ± 1.83 ng/mL, *p* < 0.05), mean sNGAL (49.82 ± 12.52 ng/mL vs. 40.57 ± 23.69 ng/mL, *p* < 0.05), mean uNGAL levels (115.40 ± 12.95 ng/mL vs. 104.95 ± 23.83 ng/mL, *p* < 0.05) and mean RRI (0.72 ± 0.05 vs. 0.69 ± 0.09, *p* < 0.05) compared to SSc patients without CRS. Conversely, we did not find a statistically significant difference in serum troponin levels between SSc patients with CRS and SSc patients without CRS (4.38 ± 2.87 ng/L vs. 3.67 ± 3.66 ng/L, *p* > 0.05).

**Figure 2:**
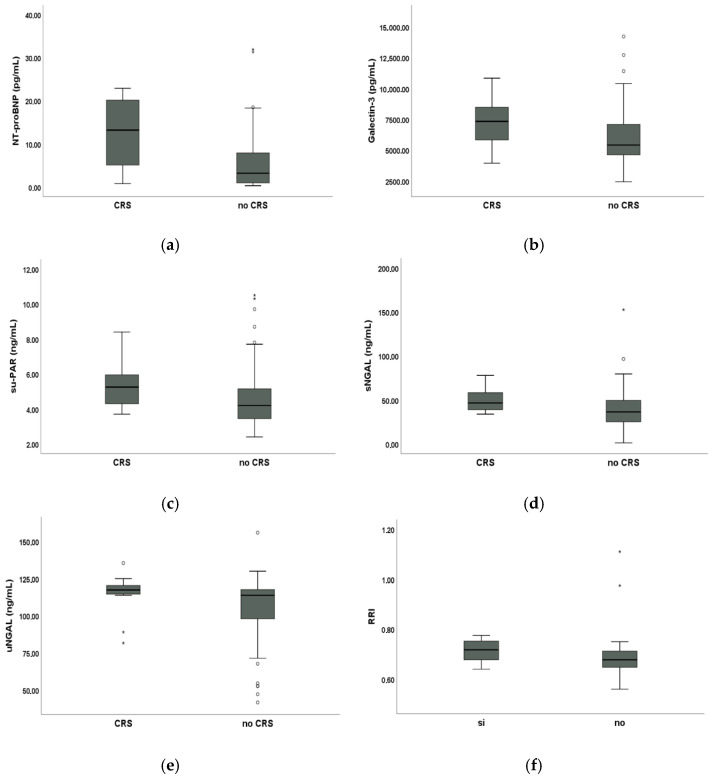
Comparative analysis of biomarkers between 16 systemic sclerosis (SSc) with cardiorenal syndrome (CRS) and 59 SSc patients without CRS: (**a**) Box plot showing median serum N-terminal pro-B-type natriuretic peptide (NT-proBNP); (**b**) box plot showing median serum galectin-3; (**c**) box plot showing median soluble urokinase plasminogen activator (suPAR); (**d**) box plot showing median serum neutrophil gelatinase-associated lipocalin (sNGAL); (**e**) box plot showing median urinary NGAL (uNGAL); (**f**) box plot showing median renal resistive index (RRI). Circles and stars are outliers.

These results are displayed in Figure 2.

In the logistic regression analysis, only NT-proBNP [OR 1.116 (CI 95%: 1.020;1.221), *p* < 0.05] was associated with CRS in SSc patients (Table 2).

## 4. Discussion

In this study, we evaluated cardiac and renal injury markers (serum/urinary NGAL, suPAR, galectin-3, NT-proBNP, troponin) in a population of 76 SSc patients and 50 HC. We also evaluated these markers in SSc patients with CRS.

Scleroderma renal vasculopathy is characterized by increased RRI and is associated with microvascular complications [19]. In SSc, RRI is a useful indicator for assessing renal involvement capable of detecting microcirculation changes due to Raynaud’s phenomenon and endothelial dysfunction. Additionally, a pathological RRI > 0.70 is a marker of fibrosis and vasculopathy in SSc. Scleroderma renal vasculopathy is one of the most common forms of subclinical renal involvement, and RRI is an early marker of damage [20]. Increased RRI is linked to microcirculatory changes, not clinically evident but long responsible for chronic kidney disease (CKD). Autopsy studies have reported intimal thickening in renal arteries and lumen narrowing, more evident in patients with SRC but also present, though less pronounced, in those without SRC [21].

In SSc patients, we previously demonstrated discrepancies between eGFR_Cr_ and eGFR_CysC_, with a lower eGFR observed when using CysC as the filtration marker [7]. Moreover, we have already found a greater statistically significant positive correlation between RRI and CysC, while only a slightly significant positive correlation between RRI and sCr, and a greater statistically significant negative correlation between RRI and eGFR_CysC_ compared to eGFR_Cr_ [7]. With this background, we defined early renal involvement by a reduction in eGFR, using serum CysC for calculation, or presence of proteinuria.

In this study, we found higher values of uNGAL, suPAR, galectin-3, NT-proBNP and troponin compared to HC. Conversely, sNGAL was lower in SSc patients than HC. This agrees with Pellicano et al. [22], who highlighted a reduction in NGAL in patients undergoing periodic iloprost infusions. Thus, in treated SSc patients, uNGAL might be more reliable.

Both serum and urinary NGAL showed on average higher trends in SSc patients with CRS; this finding is confirmed by several studies in the literature that consider NGAL a marker of both AKI and CKD [23]. Scleroderma renal vasculopathy is one of the most common forms of renal involvement in SSc patients, which could lead to ischemia/reperfusion injuries with subsequent fibrosis in affected patients [24]. According to Gharishvandi et al. [25], NGAL was superior to CysC and sCr in detecting early CKD, renal function reduction and predicting its progression.

Alvelos et al. [26] reported that measuring sNGAL concentrations in patients with acute HF and preserved renal function upon hospital admission may be a useful tool for diagnosing early CRS type 1. Nasonova et al. [27] showed that increased uNGAL concentrations were prognostic markers of declining renal function in patients with acute decompensation on chronic HF. In an experimental model of chronic HF, increased NGAL concentrations could worsen cardiac and renal dysfunctions via MMP-9 enzymatic activity and by intensifying extracellular matrix degradation [28]. Thus, NGAL may also take part in CRS type 2 pathogenesis. These studies confirm the close association between NGAL, HF and renal damage, further supporting our findings.

SSc patients with CRS showed higher suPAR levels compared to SSc patients without CRS. Increased immune activation and stimulation by inflammatory mediators induce suPAR gene expression and suPAR release; moreover, suPAR is associated with circulating neutrophils and monocytes [29]. It has been compared to classical inflammatory markers such as CRP and erythrocyte sedimentation rate (ESR), showing a stronger correlation as a marker of cellular inflammation and subclinical organ damage [29]. suPAR is associated with various SSc complications, including lcSSc, the presence of anti-Scl70, interstitial lung disease (ILD), PAH, and microvascular complications such as digital ulcers (DUs) and Raynaud’s phenomenon [30]. In patients with cardiovascular diseases, an association between elevated plasma suPAR levels with declining eGFR and the development of CKD has been demonstrated. This association was observed in patients with normal baseline renal function and was independent of conventional risk factors for kidney and cardiovascular diseases, including baseline eGFR, age, race, diabetes, hypertension, and CRP levels [31]. The linear correlation between suPAR and CysC, and between suPAR and uNGAL could strengthen the concept that these markers are independent of muscle trophism and are earlier indicators than classic renal damage methods, mainly highlighting subclinical injury. Additionally, the association with uNGAL and not with sNGAL might underline suPAR’s independence from iloprost infusions. suPAR is also a very stable molecule and is hardly influenced by other factors, including drugs [29].

Galectin-3 is characteristically overexpressed by “profibrotic” M2 macrophages and is implicated in tissue fibrosis response through sustained activation of myofibroblasts and macrophages via intracellular and extracellular signaling pathways, in a manner quite similar to SSc patients. Normal cardiac tissue expresses very little amount of galectin-3, but cardiac injury causes rapid overexpression of this marker [32]. There is an association between galectin-3 and HF, supporting its use as a tool to predict and prevent HF onset. In SSc patients, galectin-3 may be related to tissue sclerosis or aberrant angiogenesis activation characteristic of the disease. Faludi et al. [33] suggested that galectin-3 is an independent predictor of all-cause and cardiovascular mortality in SSc. Our data on galectin seem to confirm previous studies in the SSc population and in our case are even more evident in patients with CRS. Furthermore, the linear correlation between galectin-3 and suPAR, and between galectin-3 and urinary proteins may once again indicate subclinical injury, perhaps even of renal origin, that could precede classic disease manifestations.

Although NT-proBNP values remained within the normal range, SSc patients with CRS showed higher average levels compared to SSc patients without CRS. This may be due to diastolic dysfunction and cardiac overload consequent to reduced tissue elasticity, increased resistance to ventricular diastolic filling (ventricular stiffness), and increasing atrial pressures. Atrial wall stretching and conduction tissue distortion due to fibrosis provide the pathogenic substrate for rhythm and conduction disturbances and for elevated NT-proBNP [34].

In our cohort, troponin levels did not significantly differ between CRS and non-CRS patients, which may reflect the early stage of cardiac involvement in these individuals. Troponin is typically a marker of overt myocardial injury, whereas NT-proBNP is more sensitive to early subclinical changes such as diastolic dysfunction, increased ventricular stiffness, and atrial wall stress, which were more likely to be present in our population.

To our knowledge, this is the first study to comprehensively assess markers of cardiac/renal injury (serum/urinary NGAL, suPAR, galectin-3, NT-proBNP) in SSc patients with CRS.

Among the evaluated biomarkers, NT-proBNP showed the strongest association with CRS in SSc patients. However, suPAR and galectin-3, reflecting immune activation, fibrosis, and subclinical organ injury, should be considered for the evaluation of SSc patients at risk of developing CRS. Urinary NGAL, less influenced by iloprost therapy than serum NGAL, may also represent a practical tool for early detection of renal involvement. From a practical perspective, these findings suggest that combining NT-proBNP, suPAR, galectin-3, and urinary NGAL with standard clinical parameters may improve the early identification of SSc patients at higher risk of CRS. Although larger longitudinal studies are warranted, these markers could potentially be integrated into routine follow-up to support early risk stratification and guide closer monitoring.

The monocentric design of the study, the small sample size, and the absence of a prospective assessment of the markers are the main limitations of this study. Moreover, the lack of shared criteria for the diagnosis of CRS may have underestimated or overestimated the size of the examined population.

Future perspectives in evaluating CRS markers in SSc should focus on longitudinal studies, assessing these biomarkers at different time points. This would help clarify their role in the onset of cardiac and renal complications, both at diagnosis and during disease progression. Such an approach could improve early detection, refine clinical management, and ultimately enhance patient outcomes.

Finally, large, randomized studies on this topic are needed to confirm these preliminary data.

## 5. Conclusions

Poor prognosis of SSc results from the high burden of morbidity and mortality due to organ complications. PAH and cardiac damage, together with renal vasculopathy, are common visceral involvements in SSc patients. CKD is often present at the time of PAH diagnosis or may occur as a progressive decline in renal function during the course of disease. Thus, CRS may be a further complication in SSc, with a strong impact on outcome.

Considering the growing interest in the identification of specific markers to assess the diagnosis and prognosis of CRS, we demonstrated altered levels of NT-proBNP, suPAR, galectin-3, sNGAL, and uNGAL in SSc patients compared with HC. Additionally, these markers are significantly altered in SSc patients with clinically defined CRS, when compared with SSc patients without CRS.

The correlation of specific CRS markers among them confirm their role as early indicators of vasculopathy in SSc.

Further studies are needed to confirm this evidence in large longitudinal and randomized multicentric trials.

In conclusion, NT-proBNP, suPAR, galectin-3, sNGAL, and uNGAL emerge as promising biomarkers for the early detection of cardiac and renal involvement in SSc patients.

## Figures and Tables

**Table 1:** Demographic and clinical features of 76 systemic sclerosis (SSc) patients enrolled.

Variables	Mean ± Standard Deviation	Absolute Frequency (%)
Age, years	57.8 ± 10.7	
Female/male	65 (85.5)/11(14.5)	
BMI, Kg/m^2^	22.9 ± 2.8	
dcSSc/lcSSc	40 (52.6)/36 (47.4)	
Disease duration, years	15.1 ± 10.12	
mRSS	16.9 ± 9.4	
SSc-specific autoantibodies		
Anti-topoisomerase I		35 (46.1)
Anti-centromere		18 (23.7)
Anti-RNApolimerase III		5 (6.6)
Anti-Th/To		1 (1.3)
None		17 (22.4)
NVC		
Early		12 (15.8)
Active		24 (31.6)
Late		40 (52.6)
DAI	2.5 ± 1.2	
DSS	5.3 ± 2.5	
sCr, mg/dL	0.83 ± 0.19	
eGFR_Cr_, mL/min	79.82 ± 17.77	
eGFR_Cr_ ≤ 60 mL/min		12 (15.8)
sCysC, mg/L	1.12 ± 0.34	
eGFR_CysC_, mL/min	72.02 ± 20.78	
eGFR_CysC_ ≤ 60 mL/min		25 (35.9)
TAPSE/sPAP, mm/mmHg	0.80 ± 0.17	
TAPSE/sPAP ≤ 0.8 mm/mmHg		36 (48)
E/A	1.10 ± 0.44	
E/A ≤ 1		38 (50.7)
Longitudinal diameter, mm	104.66 ± 8.61	
Parenchymal thickness, mm	16.23 ± 2.49	
RRI	0.68 ± 0.05	
RRI ≥ 0.70		24 (31.6)

SSc: Systemic sclerosis. BMI: Body mass index. dcSSc: Diffuse cutaneous systemic sclerosis. lcSSc: Limited cutaneous systemic sclerosis; mRSS: Modified rodnan skin score. NVC: Nailfold videocapillaroscopy. DAI: Disease activity index. DSS: Disease severity scale. eGFR: Estimated glomerular filtration rate. sCr: Serum creatinine. sCysC: Serum cystatin. TAPSE: Tricuspid annular plane systolic excursion. sPAP: Pulmonary arterial systolic pressure. RRI: Renal resistive index.

**Table 2:** Stepwise logistic regression analysis showing the association between cardiorenal syndrome (CRS) in systemic sclerosis (SSc) patients and independent variables.

	CRS	
	OR (95% CI)	*p*
NT-proBNP, pg/mL	1.116 (1.020;1.221)	<0.05
Galectin-3, pg/mL	1.000 (1.000;1.000)	>0.05
suPAR, ng/mL	1.090 (0.754;1.575)	>0.05
sNGAL, ng/mL	0.993 (0.959;1.029)	>0.05
uNGAL, ng/mL	1.033 (0.992;1.074)	>0.05

NT-proBNP: N-terminal pro-B-type natriuretic peptide. suPAR: Soluble urokinase plasminogen activator. sNGAL: Serum neutrophil gelatinase-associated lipocalin. uNGAL: Urinary NGAL. OR: Odds ratios. CI: Confidence intervals.

## Data Availability

The original contributions presented in this study are included in the article. Further inquiries can be directed to the corresponding author.

## References

[1] Del Galdo F., Lescoat A., Conaghan P.G., Bertoldo E., Čolić J., Santiago T., Suliman Y.A., Matucci-Cerinic M., Gabrielli A., Distler O. (2024). EULAR recommendations for the treatment of systemic sclerosis: 2023 update. Ann. Rheum. Dis..

[2] Rangaswami J., Bhalla V., Blair J.E.A., Chang T.I., Costa S., Lentine K.L., Lerma E.V., Mezue K., Molitch M., Mullens W. (2019). American Heart Association Council on the Kidney in Cardiovascular Disease and Council on Clinical Cardiology. Cardiorenal Syndrome: Classification, Pathophysiology, Diagnosis, and Treatment Strategies: A Scientific Statement From the American Heart Association. Circulation.

[3] Salajova K.B., Malik J., Valerianova A. (2024). Cardiorenal Syndromes and Their Role in Water and Sodium Homeostasis. Physiol. Res..

[4] Ljungman S., Laragh J.H., Cody R.J. (1990). Role of the kidney in congestive heart failure. Relationship of cardiac index to kidney function. Drugs.

[5] Zhang X., Fishlock S., Sharpe P., McLaughlin J. Cystatin C as a biomarker for cardiorenal syndrome diseases quantitative diagnostics and monitoring via point-of-care. Proceedings of the 2022 44th Annual International Conference of the IEEE Engineering in Medicine & Biology Society (EMBC).

[6] Shlipak M.G., Sarnak M.J., Katz R., Fried L., Seliger S., Newman A., Siscovick D., Stehman-Breen C. (2005). Cystatin-C and mortality in elderly persons with heart failure. J. Am. Coll. Cardiol..

[7] Pellicano C., Carnazzo V., Basile U., Rosato E., Gigante A., Scleroderma Renal Study Group (2025). Accuracy of glomerular filtration rate estimating equations in systemic sclerosis. Eur. J. Intern. Med..

[8] Thai H.P., Bui B.H., Anh T.H., Van M.H., Raghavan S. (2020). Value of Plasma NGAL and Creatinine on First Day of Admission in the Diagnosis of Cardiorenal Syndrome Type 1. Cardiol. Res. Pract..

[9] Iacoviello M., Di Serio F., Rizzo C., Leone M., Grande D., Guida P., Gioia M.I., Parisi G., Leopizzi T., Caldarola P. (2019). Association Between High Gal-3 Serum Levels and Worsening of Renal Function in Chronic Heart Failure Outpatients. Biomark. Med..

[10] Nikorowitsch J., Borchardt T., Appelbaum S., Ojeda F., Lackner K.J., Schnabel R.B., Blankenberg S., Zeller T., Karakas M. (2020). Cardio-Renal Biomarker Soluble Urokinase-Type Plasminogen Activator Receptor Is Associated With Cardiovascular Death and Myocardial Infarction in Patients With Coronary Artery Disease Independent of Troponin, C-Reactive Protein, and Renal Function. J. Am. Heart Assoc..

[11] Van den Hoogen F., Khanna D., Fransen J., Johnson S.R., Baron M., Tyndall A., Matucci-Cerinic M., Naden R.P., Medsger T.A., Carreira P.E. (2013). 2013 classification criteria for systemic sclerosis: An American college of rheumatology/European league against rheumatism collaborative initiative. Ann. Rheum. Dis..

[12] Leroy E.C., Black C., Fleischmajer R., Jablonska S., Krieg T., Medsger T.A., Rowell N., Wollheim F. (1988). Scleroderma (systemic sclerosis): Classification, subsets and pathogenesis. J. Rheumatol..

[13] Valentini G., Iudici M., Walker U.A., Jaeger V.K., Baron M., Carreira P., Czirják L., Denton C.P., Distler O., Hachulla E. (2017). The European Scleroderma Trials and Research group (EUSTAR) task force for the development of revised activity criteria for systemic sclerosis: Derivation and validation of a preliminarily revised EUSTAR activity index. Ann. Rheum. Dis..

[14] Medsger T.A., Silman A.J., Steen V.D., Black C.M., Akesson A., Bacon P.A., Harris C.A., Jablonska S., Jayson M.I., Jimenez S.A. (1999). A disease severity scale for systemic sclerosis: Development and testing. J. Rheumatol..

[15] Cutolo M., Matucci C.M. (2007). Nailfold capillaroscopy and classification criteria for systemic sclerosis. Clin. Exp. Rheumatol..

[16] Rosato E., Gigante A., Barbano B., Cianci R., Molinaro I., Rossi C., Massa R., Amoroso A., Pisarri S., Salsano F. (2012). Intrarenal Hemodynamic Parameters Correlate with Glomerular Filtration Rate and Digital Microvascular Damage in Patients with Systemic Sclerosis. Semin. Arthritis Rheum..

[17] Ronco C., McCullough P., Anker S.D., Anand I., Aspromonte N., Bagshaw S.M., Bellomo R., Berl T., Bobek I., Cruz D.N. (2009). Cardio-renal syndromes: Report from the consensus conference of the Acute Dialysis Quality Initiative. Eur. Heart J..

[18] Colalillo A., Grimaldi M.C., Vaiarello V., Pellicano C., Leodori G., Gigante A., Romaniello A., Rosato E. (2021). In systemic sclerosis, the TAPSE/sPAP ratio can be used in addition to the DETECT algorithm for pulmonary arterial hypertension diagnosis. Rheumatology.

[19] Scheen M., Dominati A., Olivier V., Nasr S., De Seigneux S., Mekinian A., Issa N., Haidar F. (2023). Renal involvement in systemic sclerosis. Autoimmun. Rev..

[20] Bruni C., Rosato E., Maestripieri V., Gigante A., Tesei G., Bellando-Randone S., Guiducci S., Chiostri M., El Aoufy K., Blagojevic J. (2019). The Renal Resistive Index in systemic sclerosis: Determinants, prognostic implication and proposal for specific age-adjusted cut-offs. Eur. J. Intern. Med..

[21] Trostle D.C., Bedetti C.D., Steen V.D., Al-Sabbagh M.R., Zee B., Medsger T.A. (1988). Renal vascular histology and morphometry in systemic sclerosis. a case–control autopsy study. Arthritis Rheum..

[22] Pellicano C., Colalillo A., De Marco O., Carnazzo V., Basile U., Gigante A., Cianci R., Rosato E. (2024). Iloprost infusion reduces serological cytokines and hormones of hypoxia and inflammation in systemic sclerosis patients. Clin. Exp. Med..

[23] Vanmassenhove J., Vanholder R., Nagler E., Van Biesen W. (2012). Urinary and serum biomarkers for the diagnosis of acute kidney injury: An in-depth review of the literature. Nephrol. Dial. Transplant..

[24] Gigante A., Leodori G., Pellicano C., Villa A., Rosato E. (2022). Assessment of kidney involvement in systemic sclerosis: From scleroderma renal crisis to subclinical renal vasculopathy. Am. J. Med Sci..

[25] Gharishvandi F., Kazerouni F., Ghanei E., Rahimipour A., Nasiri M. (2015). Comparative Assessment of Neutrophil Gelatinase-Associated Lipocalin (NGAL) and Cystatin C as Early Biomarkers for Early Detection of Renal Failure in Patients with Hypertension. Iran. Biomed. J..

[26] Alvelos M., Pimentel R., Pinho E., Gomes A., Lourenço P., Teles M.J., Almeida P., Guimarães J.T., Bettencourt P. (2011). Neutrophil Gelatinase-Associated Lipocalin in the Diagnosis of Type 1 Cardio-Renal Syndrome in the General Ward. Clin. J. Am. Soc. Nephrol..

[27] Nasonova S.N., Zhirov I.V., Ledyakhova M.V., Sharf T.V., Bosykh E.G., Masenko V.P., Tereshchenko S.N. (2019). Early diagnosis of acute renal injury in patients with acute decompensation of chronic heart failure. Ter. Arkhiv.

[28] Angelini A., Castellani C., Virzì G.M., Fedrigo M., Thiene G., Valente M., Ronco C., Vescovo G. (2015). The Role of Congestion in Cardiorenal Syndrome Type 2: New Pathophysiological Insights into an Experimental Model of Heart Failure. Cardiorenal Med..

[29] Rasmussen L.J.H., Petersen J.E.V., Eugen-Olsen J. (2021). Soluble Urokinase Plasminogen Activator Receptor (suPAR) as a Biomarker of Systemic Chronic Inflammation. Front. Immunol..

[30] Vasarhelyi B., Toldi G., Balog A. (2016). The Clinical Value of Soluble Urokinase Plasminogen Activator Receptor (suPAR) Levels in Autoimmune Connective Tissue Disorders. Electron. J. IFCC.

[31] Spinale J.M., Mariani L.H., Kapoor S., Zhang J., Weyant R., Song P.X., Wong H.N., Troost J.P., Gadegbeku C.A., Gipson D.S. (2015). A reassessment of soluble urokinase-type plasminogen activator receptor in glomerular disease. Kidney Int..

[32] Dong R., Zhang M., Hu Q., Zheng S., Soh A., Zheng Y., Yuan H. (2018). Galectin-3 as a novel biomarker for disease diagnosis and a target for therapy (Review). Int. J. Mol. Med..

[33] Faludi R., Nagy G., Tőkés-Füzesi M., Kovács K., Czirják L., Komócsi A. (2017). Galectin-3 is an independent predictor of survival in systemic sclerosis. Int. J. Cardiol..

[34] Valentini G., Vitale D., Giunta A., Maione S., Gerundo G., Arnese M., Tirri E., Pelaggi N., Giacummo A., Tirri G. (1996). Diastolic abnormalities in systemic sclerosis: Evidence for associated defective cardiac functional reserve. Ann. Rheum. Dis..

